# Tracking the Distribution of *Brucella abortus* in Egypt Based on Core Genome SNP Analysis and In Silico MLVA-16

**DOI:** 10.3390/microorganisms9091942

**Published:** 2021-09-13

**Authors:** Katharina Holzer, Mohamed El-Diasty, Gamal Wareth, Nour H. Abdel-Hamid, Mahmoud E. R. Hamdy, Shawky A. Moustafa, Jörg Linde, Felix Bartusch, Ashraf E. Sayour, Essam M. Elbauomy, Mohamed Elhadidy, Falk Melzer, Wolfgang Beyer

**Affiliations:** 1 Department of Livestock Infectiology and Environmental Hygiene, Institute of Animal Science, University of Hohenheim, Garbenstraße 30, 70599 Stuttgart, Germany; wolfgang.beyer@uni-hohenheim.de; 2 Agricultural Research Center, Animal Health Research Institute, P.O. Box 264-Giza, Cairo 12618, Egypt; dr_mesbah_m@yahoo.com (M.E.-D.); nour78_78@yahoo.com (N.H.A.-H.); merhamdy@hotmail.com (M.E.R.H.); sayourashraf@gmail.com (A.E.S.); elbauomyessam@yahoo.com (E.M.E.); 3 Faculty of Veterinary Medicine, Benha University, Moshtohor, Toukh 13736, Egypt; gamal.wareth@fli.de (G.W.); dr.shawky.gesriha@gmail.com (S.A.M.); 4 Institute of Bacterial Infections and Zoonoses, Friedrich-Loeffler Institut, Naumburger Str. 96a, 07743 Jena, Germany; joerg.linde@fli.de (J.L.); falk.melzer@fli.de (F.M.); 5 High-Performance and Cloud Computing Group, IT Center (ZDV), University of Tuebingen, Waechterstrasse 76, 72074 Tübingen, Germany; felix.bartusch@uni-tuebingen.de; 6 Biomedical Sciences Program, University of Science and Technology, Zewail City of Science and Technology, October Gardens, 6th of October City, Giza 12578, Egypt; melhadidy@zewailcity.edu.eg; 7 Department of Bacteriology, Mycology and Immunology, Faculty of Veterinary Medicine, Mansoura University, Mansoura 35516, Egypt

**Keywords:** *Brucella abortus*, SNP analysis, genotyping, MLVA, outbreak analysis

## Abstract

Brucellosis, caused by the bacteria of the genus *Brucella*, is one of the most neglected common zoonotic diseases globally with a public health significance and a high economic loss among the livestock industry worldwide. Since little is known about the distribution of *B. abortus* in Egypt, a total of 46 *B. abortus* isolates recovered between 2012–2020, plus one animal isolate from 2006, were analyzed by examining the whole core genome single nucleotide polymorphism (cgSNP) in comparison to the in silico multilocus variable number of tandem repeat analysis (MLVA). Both cgSNP analysis and MLVA revealed three clusters and one isolate only was distantly related to the others. One cluster identified a rather widely distributed outbreak strain which is repeatedly occurring for at least 16 years with marginal deviations in cgSNP analysis. The other cluster of isolates represents a rather newly introduced outbreak strain. A separate cluster comprised RB51 vaccine related strains, isolated from aborted material. The comparison with MLVA data sets from public databases reveals one near relative from Argentina to the oldest outbreak strain and a related strain from Spain to a newly introduced outbreak strain in Egypt. The distantly related isolate matches with a strain from Portugal in the MLVA profile. Based on cgSNP analysis the oldest outbreak strain clusters with strains from the UK. Compared to the in silico analysis of MLVA, cgSNP analysis using WGS data provides a much higher resolution of genotypes and, when correlated to the associated epidemiological metadata, cgSNP analysis allows the differentiation of outbreaks by defining different outbreak strains. In this respect, MLVA data are error-prone and can lead to incorrect interpretations of outbreak events.

## 1. Introduction

Brucellosis is a globally distributed zoonotic disease caused by bacteria of the genus *Brucella*, infecting mammals including humans [1]. Out of 12 species, *B. melitensis*, *B*. *abortus*, and *B. suis* are considered to be the most important human pathogens with varying pathogenicity [2,3]. *B. melitensis* and *B. suis* except biovar 2, are the most virulent species, whereas *B. abortus* provokes milder illness [4]. Small ruminants and bovines are the predominant hosts for *B. melitensis* and *B. abortus* respectively, while cross species transmission has been proven [5,6]. In a recent study *B. abortus* was found in atypical carriers like cats and dogs [7]. Interspecies infection [7,8] and host spill over can be explained by keeping different animals and animal waste in close contact. Since centuries, *Brucella* affects the public health and the economy of several countries leading to a serious threat to human health and substantial economic losses, especially in developing countries [9]. Humans acquire the disease through contact with infected animals and consumption of their products like unpasteurized milk being the most common source of infection in urban populations [10,11]. In Egypt, *B. melitensis* biovar 3 is responsible for most human and animal cases, followed by *B. abortus* biovar 1 [12,13]. Although the World Health Organization (WHO) and the World Organization for Animal Health (OIE) have recommended strategies and measures to control or even eradicate brucellosis, only several countries in Europe, Canada, Japan, Australia and New Zealand are believed to be free from brucellosis [14]. The epidemiological situation in the Middle East, in the Mediterranean basin, some parts of central and south America, Africa and Asia is alarming [11].

Despite the implementation of brucellosis control program in 1981 [12], brucellosis is still endemic in Egypt. The incidence has increased steadily since 1960 when Friesian cows were imported [12]. Since then, the disease has been recognized as one of the most important livestock diseases in the country. The annual incidence ranges from 64 to 70 per 100.000 human population [15]. Reasons are low biosafety levels in farms, high livestock and human density, traditional food habits and limited success of the official control program [16]. The movement of animals for grazing or trade further enhances the spread of the disease throughout the country [17].

Proper brucellosis control requires surveillance and highly discriminatory methods to determine the source of infection and spread of outbreak strains. Multilocus Variable Number of Tandem Repeat Analysis (MLVA) has been used for genotyping but cannot accurately trace the origin and transmission of *Brucella* outbreak strains [18].

In recent years, typing methods have shifted towards whole genome-based approaches like analysis of single nucleotide polymorphisms (SNPs) that allow both a more in-depth resolution of genotypes [19] and subsequent phylogenetic correlations as well.

While the knowledge about the distribution of *B. melitensis* in Egypt is growing, epidemiological data about the diversity and distribution of *B. abortus* is scarce. So far, and to the best of our knowledge, twenty-six *B. abortus* isolates from Egypt were analyzed by lab-based MLVA-16 [20,21] and two *B. abortus* isolates by MLVA-15 [22]. Analysis of eight *B. abortus* isolates by WGS was published recently [23].

Herewith, the diversity, phylogeographic distribution and possible spread of outbreak strains of *B. abortus* in Egypt from 2012 to 2020 were investigated including one isolate from 2006, by using both WGS based cgSNP analysis and MLVA.

## 2. Materials and Methods

### 2.1. Origin of Bacterial Isolates

A total of 46 *B. abortus* isolates were collected in 2012–2020 plus one isolate in 2006 (HEA68, Beni Suef) from both animals and humans from various Egyptian governorates, in the north and one in the south (isolate 21906, Aswan, 2020), which were previously reported to be endemic areas of brucellosis. For the purpose of epidemiological interpretation of genotyping data any isolation of *B. abortus* from human or animal specimens, including the ten milk samples, is considered here as an outbreak and the isolate is termed an “outbreak strain”.

Two human samples were obtained from blood of *Brucella* infected patients admitted to hospitals for specialized cases (no patient data was involved in this study). The remaining 45 samples were taken from animals, including buffaloes (four isolates), cattle (17), cows (18), sheep (4), one isolate from a cat, one isolate from a dog and additionally, two purchased *B. abortus* RB51 strains from vaccine batches, purchased from CZ Veterinaria, Spain, were available. Among these isolates from animals, ten isolates were directly recovered from milk (five from cows and five from cattle, both defined as cows from now on), five from fetal abomasal contents (all from cows), one from fetal liver (cow), one from lung (cow), three from lymph nodes (two from buffaloes and one from a sheep), two from unspecified organs (cows), one from the placenta (cow), one from retropharyngeal lymph nodes (buffalo), four from spleen (one from an ewe and three from cows), one from stomach contents (cow), one from stomach contents of an aborted fetus, six from supramammary lymph nodes (one from a buffalo, two from cows and three from ewes) and nine from uterine discharge (seven from cows, one from a cat and one from a dog). All these animal isolates originate from different animals. All isolates and both vaccine batches with their metadata are listed in Table S1.

The Animal Health Research Institute (AHRI) ethical committee in Giza approved the study (ethical code Ref. No. 165716). The consent of the patients was sought. Written informed consent was obtained from all participants. All patient data were blinded.

### 2.2. Brucella Isolation from Sample Materials and DNA Extraction

Swab samples were smeared on *Brucella* selective agar (Oxoid GmbH, Wesel, Germany) containing 2500 IU Polymyxin B, 12500 IU Bacitracin, 2.5 mg Nalidixic acid, 50,000 IU Nystatin, 10 mg Vancomycin and 25 mg Natamycin in a total volume of 500 mL *Brucella* selective agar and incubated at 37 °C in the presence of 5–10% CO_2_ for at least five days. Isolates were passaged three times to obtain pure *Brucella* colonies. Single colonies or streaks of it from selective agar medium were picked from selective agar medium and grown in Brucella broth for around three days for DNA extraction. DNA was extracted using Qiagen DNeasy Blood & Tissue Kits (Qiagen, Hilden, Germany) according to the manufacturer’s guidelines. The DNA of six isolates was extracted directly after cultivating and the remaining 41 isolates and both vaccine isolates were heat inactivated before.

### 2.3. Whole Genome Sequencing and Bioinformatics Procedure of the Raw Reads

Default genomic library preparation and total genomic DNA sequencing were performed by Eurofins Genomics GmbH (Konstanz, Germany). The libraries were sequenced using Illumina Novaseq 6000, producing at least 5 million 151 bp paired-end reads. High-throughput shortread sequencing yielded an average of 12,176,974 reads per isolate (min 3,931,960, max 17,149,918), leading to a mean coverage of 553 (min 178, max 780). For analyzing the WGS data in a standardized and automated manner the Linux-based bioinformatics WGSBAC (v.2.1) pipeline (https://gitlab.com/FLI_Bioinfo/WGSBAC/-/tree/version2, accessed on 2 April 2021) was used. The pipeline input consisted of a metadata file and the Illumina paired-end fastq files. Raw coverage for each dataset was calculated by the number of reads multiplied by their average read length and divided by the genome size. Contigs were assembled using Shovill v.1.0.4 (https://github.com/tseemann/shovill, accessed on 24 April 2021), an optimizer for SPAdes assembler [24]. Quality control of the assembled contigs was performed by QUAST v.5.0.2 [25]. QC data are provided in Table S2. All fastq files were submitted to the National Center for Biotechnology Information (NCBI) under the BioProject number PRJNA742519 (https://www.ncbi.nlm.nih.gov/bioproject/742519, accessed on 9 September 2021).

### 2.4. Species Determination

The species of isolates was determined by Bruce-ladder PCR in silico using the program Geneious v.11.1.5 based on assembled contigs as calculated by the program Shovill (https://github.com/tseemann/shovill, accessed on 24 April 2021) in the bioinformatics pipeline. As an exception the 2524 bp fragment, which represents one of the four fragments specific for the *B. abortus* RB51 vaccine, was determined by lab-based single fragment Bruce-ladder PCR, as it could not be detected in silico (Figure S4).

The Bruce-ladder PCR-based fragment analysis [26,27,28] was performed as follows: After a polymerase dependent first heating at 95 °C, 29 cycles were run, comprising of: Heating at 94 °C for 45 s, annealing at 59 °C for 90 s and elongation at 72 °C for three min followed by 72 °C for ten min. The primers used are listed in Table S3.

### 2.5. Core Genome SNP Genotyping

In silico Single Nucleotide Polymorphism (SNP) calling was performed using Snippy (v.4.6.0) with default parameters (https://github.com/tseemann/snippy, accessed on 24 April 2021). Calculation of SNPs was done on the core genome without rRNA genes. cgSNPs (Table S4) were called based on alignment to the *B. abortus* 2308 reference strain (GenBank accession numbers NC_007618.1 and NC_007624.1). Additionally, cgSNP calling was also performed based on the *B. abortus* vaccine strain RB51 (GenBank accession numbers NZ_CP046720.1 and NZ_CP046721.1) set as a reference to test for cgSNP differences to the purchased vaccine batches and vaccine like isolates (Table S5). Core genome SNP based genotypes (cgSNPGTs) were defined using maximum one cgSNP difference.

### 2.6. Calculation of Trees and Simpson’s Diversity Index

Cluster analysis, phylogenetic analysis, creation of minimum spanning trees (MST) and the determination of the Simpson’s diversity index (SDI) were performed with Bionumerics version 8.0 (Applied Maths, Sint-Martens-Latem, Belgium). One SNP and one Bruce marker were used to define a genotype for SDI. For the cgSNP dendrogram, a maximum parsimony (MP) tree was created based on the character data. For MLVA, a neighbor-joining (NJ) tree based on categorical data was created. Both trees were permutated 1000 times and rooted by maximum branch length. The MSTs are presented with logarithmic scaling. For a better representation, Figure 2 was modified with the program Adobe Acrobat Pro 2017 (Adobe Systems Software Ireland Limited, Dublin, Ireland).

### 2.7. In Silico and Lab-Based MLVA

Multilocus Variable Number of Tandem Repeats (MLVA-16) genotyping was carried out in silico using MISTReSS (https://github.com/Papos92/MISTReSS, accessed on 24 April 2021) with primers adapted for *Brucella* (Table S3) [29]. To avoid multiple primer binding sites the sequence of the forward primer of Bruce21 was extended to (5′-GGCAGTGGGGCAGTGAAGAATATGGTCGCTGCGCTCATGCGCAACCAAA-ACA-3′).

For validation purpose, the MLVA-16 output data from the bioinformatics pipeline were compared with lab-based MLVA using fragment based capillary electrophoresis according to Le Fleche et al. (2006) [30] with the modifications by Al Dahouk et al. (2007) [31] in an ABI3130 instrument with POP7 Polymer (Advanced Biolab Service Gesellschaft fuer Laborgeraete, Beratung und Support mbH, Munich, Germany) and GeneScan™ 1200 LIZ™ dye (ThermoFisher Scientific, Dreieich, Germany) as the size standard. The number of repeats at each locus was determined by the amplicon size according to the published *Brucella* allele assignment table [31]. All MLVA profiles have been submitted to the MLVA database (https://microbesgenotyping.i2bc.paris-saclay.fr/databases/view/40, accessed on 21 May 2021).

To further check the reliability of the MLVA results provided by the bioinformatics pipeline described in Section 2.5 and lab-based MLVA, 83 MLVA code strings from *B. melitensis* and *B. abortus* isolates were compared. Thereof, a total of 65 PCR-based MLVA code strings of the used isolates were already published by Wareth et al. [20]. In total, 1328 fragment lengths of the VNTR-amplicons were used for the comparison. In case of deviations between both methods, the amplicon was cloned into a plasmid vector and sequenced by the Sanger technique to determine the correct fragment length. MLVA based genotypes (MLVAGTs) were defined using maximum one marker difference. All in silico MLVA results of the mentioned isolates in Section 2.1, are listed in Table S6.

### 2.8. Comparison with Entries in Public Databases

In order to compare the 47 *B. abortus* isolates and the both vaccine batches, as described in Section 2.1, entries of *B. abortus* strains were taken from the public databases and processed bioinformatically as described in Section 2.5. First, to ensure that the reads downloaded from Sequence Read Archive (SRA) (https://www.ncbi.nlm.nih.gov/sra, accessed on 19 June 2021) and all assemblies downloaded from Genbank (https://www.ncbi.nlm.nih.gov/genbank/, accessed on 24 April 2021) originate from *B. abortus* isolates, the average nucleotide identity (ANI) to the reference (*B. abortus*, 2308) was computed. For the reads downloaded from SRA, the corresponding assemblies calculated by Shovill (https://github.com/tseemann/shovill, accessed on 24 April 2021) were used. The tool pyANI v. 0.2.10 (https://github.com/widdowquinn/pyani#conda, accessed on 19 June 2021) [32] was used to compute a pairwise ANI and other metrics between all assemblies. MUMmer (NUCmer) v. 3.23 (https://github.com/mummer4/mummer, accessed on 19 June 2021) [33] was used by pyANI to align the input sequences. For ongoing analysis at least 99% nucleotide identity was accepted when aligned to *B. abortus* 2308 reference strain.

Since our cgSNP analysis is based on the program Snippy (https://github.com/tseemann/snippy, accessed on 24 April 2021), which uses the raw Illumina reads for analysis, just 363 available *B. abortus* data sets with known origin could be used. The appropriate cgSNP distance table comprising 413 data sets in total (with the reference strain *B. abortus* 2308) is provided in Table S7.

One hundred fifty-nine sequence assemblies of *B. abortus* entries with known origin were downloaded from the GenBank database. In total, 478 entries from GenBank and SRA could be used for MLVA. The list of MLVA-strings is provided in Table S8.

All entries of the isolates from both databases, GenBank and SRA, and their metadata are provided in Table S9.

### 2.9. Geographical Map

The geographical map was created using the Free and Open Source QGIS version 3.12.2 Bucuresti (https://www.qgis.org/de/site/forusers/download.html, accessed on 9 September 2021), generated from GPS data in Google Maps and the layer EPSG: 4326 and WGS: 84. The appropriate QGIS download file is available in the Supplementary Data.

## 3. Results

### 3.1. Origin of Bacterial Isolates and Their Classification

**Figure 1:**
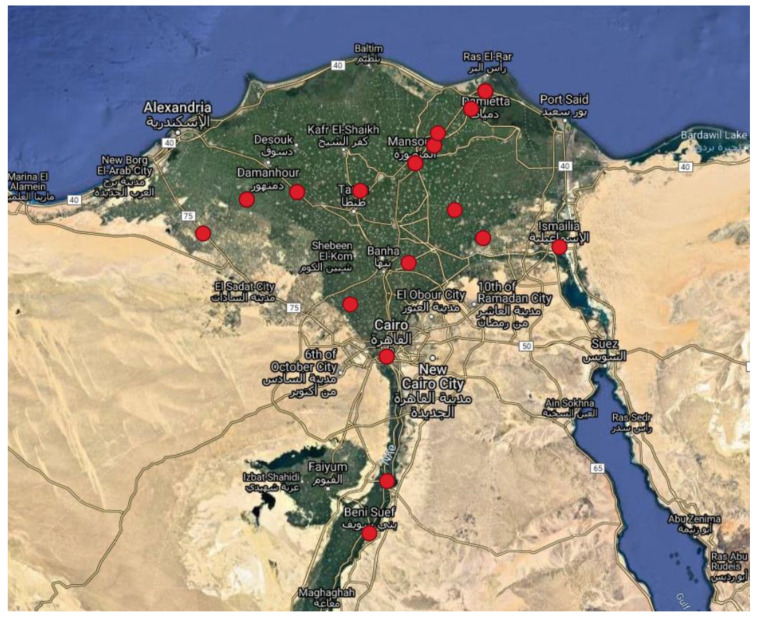
Geographic origin of *B. abortus* isolates from the North of Egypt. In this map the isolates in the associated governorates are shown. If the city within the governorate was available in the metadata (Table S1), the more precise geographic location of the city was taken. Otherwise, the geographic location of the middle of the governorate was taken. The single isolate from Aswan, south of Egypt, refers to 21,906 (not shown in the map). This interactive map was built with QGIS v.3.12.2 and can be downloaded from the Supplementary Material.

Isolates included in this study were obtained from 11 Egyptian governorates over a period of 15 years. Figure 1 shows the place of sampling, mainly in the northern part of Egypt. An interactive GIS-Map based on QGIS 3.12.2 was established, allowing for geographical mapping of the isolates with all available metadata (QGIS File in the Supplements).

Among the two vaccine batches and 47 outbreak related isolates of *Brucella*, collected from human and six animal species, 40 were specified as *B. abortus* and nine as RB51 vaccine by in silico Bruce-ladder PCR with support by lab-based PCR analysis of the 2524 bp fragment for RB51 (Figure S4). Besides the vaccine batches 5842 and 5843 the following isolates were classified as RB51 related strains: 5261, 15646, HEA15, HEA16, HEA23, HEA30 and HEA45.

### 3.2. Core Genome SNP Genotyping for B. abortus

**Figure 2:**
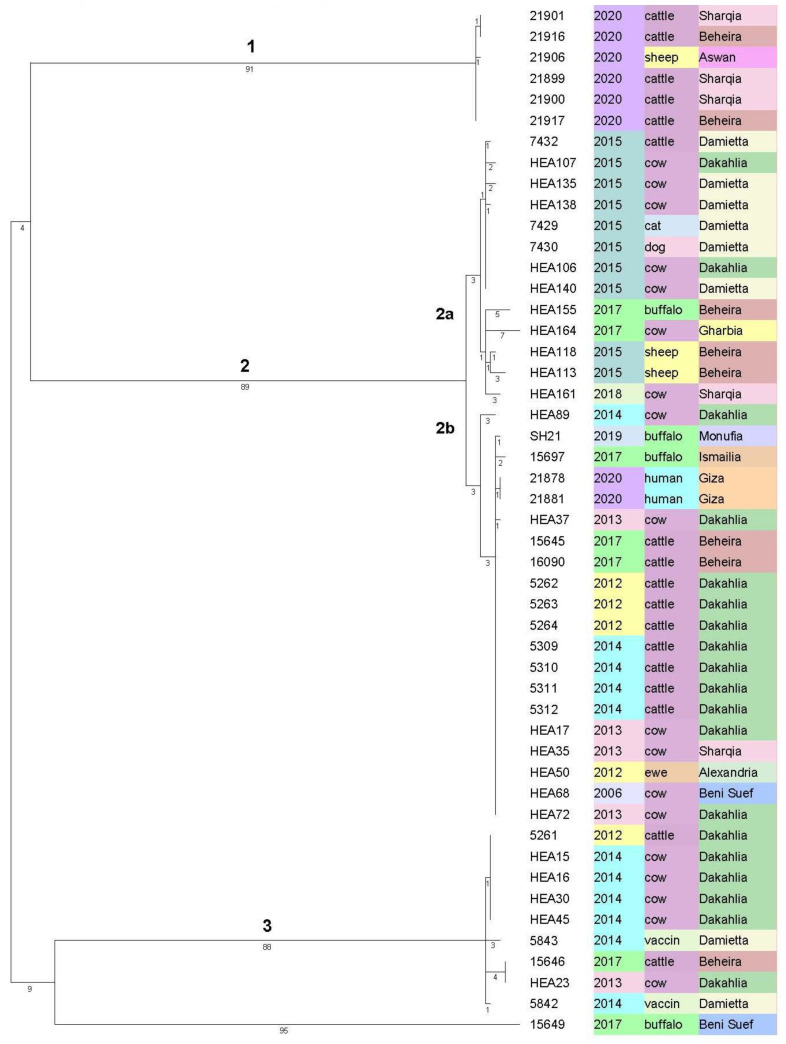
Maximum Parsimony tree for the 45 *B. abortus* field isolates plus two human isolates and two vaccine batches. The tree is based on WGS core genome SNP analysis and was calculated in Bionumerics with 1000 permutations to get the highest resampling support and rooted by maximum branch length. *B. abortus* 2308 was set as reference. The three columns represent the year of isolation, the host and the governorate, correspondingly. This automatically generated picture from Bionumerics has been modified for a better representation by Adobe Acrobat Pro 2017.

**Figure 3:**
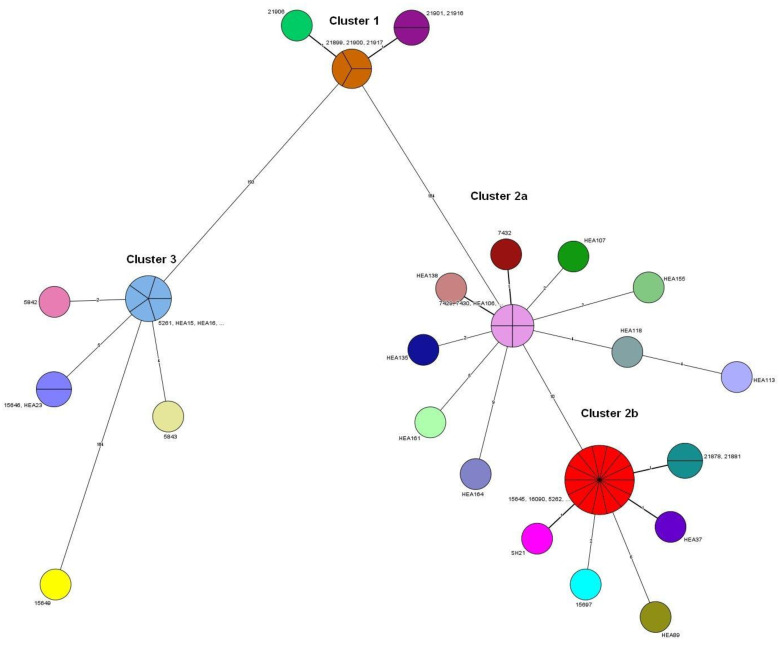
Minimum spanning tree (MST) for the 45 *B. abortus* field isolates plus two human isolates and two vaccine batches. The tree is based on cgSNP analysis provided by Snippy and was calculated in Bionumerics. The same color represents the same genotype.

Based on cgSNP analysis of WGS-data (Figure 2 and Figure 3) the isolates are ordered in three main clusters comprising a total of 24 cgSNP genotypes (SNPGT). The Simpson’s Diversity Index (SDI) based on one cgSNP distance is 0.9. The branch lengths between clusters 1 and 2, clusters 1 and 3 and clusters 2 and 3 are 184, 193 and 195 respectively. Isolate 15649 represents an outlier with 184 cgSNPs distance to the closest cluster 3. The cgSNP distances are provided in Table S4.

Cluster 1 comprises six isolates from sheep and cattle. All were isolated in 2020. Three isolates share the same cgSNPGT while three more isolates differ in one cgSNP from the former three. Five of these isolates are from Beheira and Sharqia (170 km distance) in northern Egypt. Isolate 21906 from sheep was found in Aswan (south of Egypt), about 1000 km from Beheira.

Cluster 2 can be divided into two subclusters (SC) 2a and 2b, differing by a total of 10 cgSNPs in the MST analysis (Figure 3). SC2a consists of two groups of isolates. Eight isolates from Damietta and Dakahlia, among them those from a cat and a dog, were all isolated in 2015 and differ in up to two cgSNPs only (Figure 2). The isolates of the second group are more diverse. Five isolates were collected from three different locations (HEA113 and HEA118 from Beheira in 2015, HEA155 and HEA164 from Beheira and Gharbia in 2017, and HEA161 from Sharqia in 2018) and differ in up to twelve cgSNPs to each other. The largest distance shows isolate HEA164 from a cow in Gharbia, 2017.

In SC2b the largest group, which is very homogenous and differing in two cgSNPs maximum, consists of 14 isolates with exactly the same cgSNPGT. They have been isolated from five different governorates, once in 2006 (HEA68) and again over a period of six years (2012 to 2017) from cows and one ewe. Very close to this group are both the human isolates from Giza in 2020 differing in one cgSNP only and hence are considered as the same outbreak strain. There are two more highly similar isolates, SH21 from buffalo in Monufia (2019) and HEA37 from a cow in Dakahlia (2013), differing also in just one cgSNP, plus one isolate 15697 from Ismailia (buffalo, 2017) differing in two cgSNPs and shown as descendants from the largest group in the MST (Figure 3). There is one isolate from Dakahlia, 2014 (HEA89) which differs in six cgSNPs from the large group in SC2b (15645-HEA72).

The entire 3rd cluster represents isolates genetically highly related to the vaccine strain RB51. Five isolates from Dakahlia (2012 and 2014) have an identical cgSNPGT, with five cgSNPs difference to two other isolates from Dakahlia and Beheira in 2013 and 2017. With the exception of one isolate (HEA23) cultured from the spleen of a slaughtered cow, all isolates were taken from aborted materials in the field. For the purpose of comparison, isolates from two RB51 vaccine batches (5842 and 5843 in Figure 2) were included in the analysis. These both vaccine batches show a difference of four cgSNPs to one another and are related to the field isolates by a distance between two and seven cgSNPs in total. The greatest distance of seven cgSNPs was found between vaccine 5843 and HEA23/15646. Running a Bruce-ladder PCR in silico also classified the field isolates as RB51 vaccine strains. To further support this classification, the analysis of the raw data was repeated with the reference strain RB51. The MP analysis located the RB51 reference strain also in cluster 3 (data on request from author K.H.) with one cgSNP difference to vaccine 5842 and three cgSNPs difference to vaccine 5843 (Table S5).

### 3.3. Comparing B. abortus cgSNP Analysis to MLVA Genotyping

**Figure 4:**
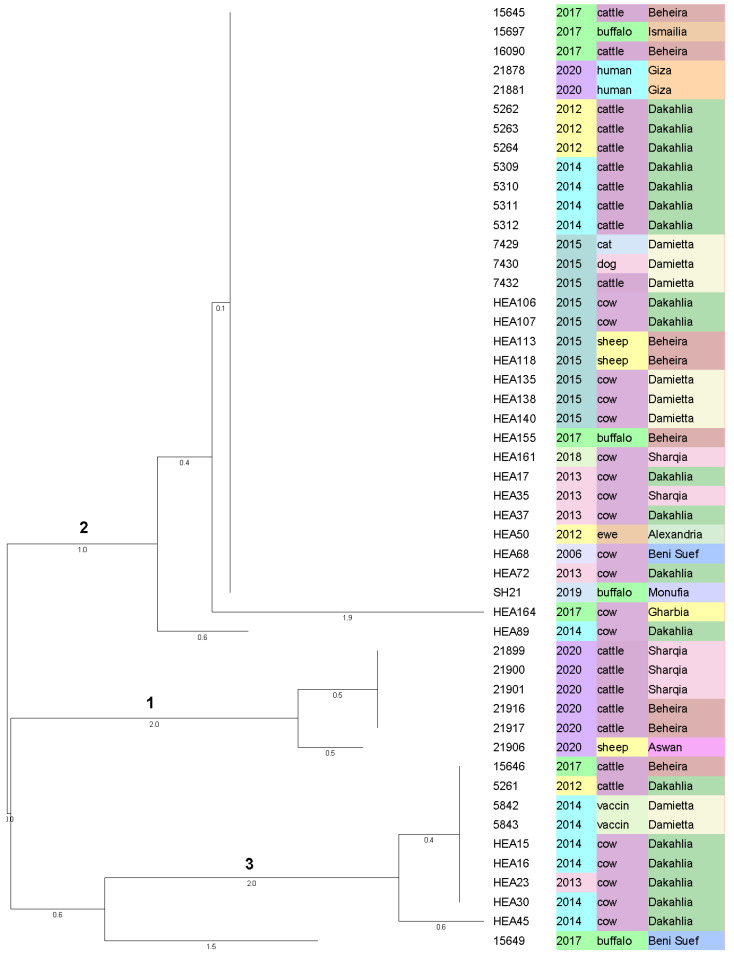
Neighbor-joining (NJ) tree for the 45 *B. abortus* field isolates plus two human isolates and two vaccine batches. The tree is based on MLVA-16 data from MISTReSS (https://github.com/Papos92/MISTReSS, accessed on 24 April 2021), calculated in Bionumerics using categorical values with 1000 permutations to receive the highest resampling support. The root position was set as the maximum branch length. The three columns represent the year of isolation, the host and the governorate, correspondingly.

Based on in silico analysis of 16 marker-MLVA, performed on the WGS data using MISTReSS, the isolates are ordered in three main clusters comprising a total of eight MLVA genotypes (MLVAGT) (Figure 4 and Figure S1). Cluster numbering is used in relation to the clusters in Figure 2. The SDI based on one marker distance is 0.56.

Cluster 1 consists of a group of five isolates with identical MLVAGT which had been separated by cgSNP analysis into two genotypes but with only one cgSNP difference. Isolate 21906 from Aswan in the south of Egypt represents a different genotype in both analyzes with one marker and one cgSNP difference, respectively, to the other isolates of this cluster.

Cluster 2 comprises 33 isolates, 31 of them with identical MLVAGT including both human isolates. These 31 isolates had been discriminated in 16 different SNPGTs by cgSNP analysis belonging to SNP subclusters 2a and 2b, respectively. Isolates HEA164 and HEA89 represent single MLVAGTs with two and one different markers. Both isolates were also clearly separated by up to 13 and up to eight cgSNPs from their respective subclusters.

Cluster 3 comprises nine isolates with identical MLVAGT representing the RB51 related group of strains, among them two vaccine batches. The cgSNP analysis separated this group into four SNPGTs where HEA45 is identical to four other isolates. In the MLVA, however, HEA45 is separated by one marker from the rest.

Isolate 15649 represents a highly different MLVAGT with four markers difference to both cluster 1 and cluster 3. This is in accordance with the long branch distance of 183 cgSNPs to the nearest cluster 3.

By comparison with the MLVA database for *Brucella* isolates, there are five new MLVAGTs found in our panel of *B. abortus*. All new MLVAGTs are from Cluster 1 and 2. The other MLVAGTs have been described for isolates from the USA, Brazil, UK and Costa Rica. All MLVA results are provided in Table S8.

### 3.4. Strain Comparison with Public Database Entries

To compare the isolates presented in this study with available entries from GenBank and the NCBI Sequence Read Archive (SRA), a cgSNP analysis (Figure S2) and a MLVA (Figure S3) were performed. For cgSNP analysis 363 datasets from SRA database were available and for MLVA 478 datasets from GenBank database (see Section 2.8 for explanation). The entire cgSNP analysis based on cgSNP distances is provided in Table S7. The cgSNP analysis reveals that isolates from cluster 2 in Figure 2 are with ten cgSNPs related to database entries from the UK while isolates from cluster 1 (Figure S2) and the single isolate 15649 has no near relatives. According to the MLVA there is a near relative (one marker difference) from Argentina, isolated from human in 2004, to the isolates of cluster 2 (Figure S3). Other entries from USA and UK differ in two markers to cluster 2, among them another isolate from Argentina. One entry from Spain, differing in two markers, is closest to the isolates in cluster 1 (Figure S3). The single isolate 15649 has the same MLVAGT as one strain from Portugal isolated in 2006 from a goat (Figure S3). Other entries from the USA, UK, Argentina and India differ with one marker to 15649.

### 3.5. Comparison of the MLVA Results of PCR Based Fragment Analysis and In Silico Analysis

To the validation purpose, a comparison of fragment length and deduced copy numbers between the MLVA results drawn from lab-based PCR fragment analysis and in silico MLVA analysis was performed for 83 datasets of *B. abortus* and *B. melitensis* (data on request from author K.H.). Among the latter were lab-based PCR fragment data published by Wareth et al. [20]. In total, 35 deviations between copy numbers were found. Among them were six deviations in Bruce06, three deviations in Bruce09, two deviations in Bruce16 and 24 deviations in Bruce19. Second, our MLVA results based on PCR fragment length analysis were compared to those results based on in silico analysis. Two deviations in the Bruce06 marker could be determined. After repeating those PCR fragment analyzes in the lab and sequencing some of the PCR-fragments in question all copy numbers deduced were in full accordance with the results taken from the in silico bioinformatics analysis.

## 4. Discussion

While the distribution of *B. melitensis* in Egypt has been analyzed based on PCR fragment length polymorphism of 15 or 16 so-called VNTR markers [20,21,22,34], epidemiological information of the spread and distribution of *B. abortus* in Egypt is very scarce. Appropriately, we focused our analyzes here on the distribution and possible spread of outbreak strains of *B. abortus* using a panel of 47 isolates collected from 11 governorates of the entire northern part of Egypt, with the exception of one single isolate from southern Egypt. Forty-six of our isolates were collected from 2012–2020 and one isolate is from 2006. Additionally, two isolates from RB51 vaccine batches were included. Unfortunately, no isolates from the years 2007–2010 were available for this study.

Data from in silico genotyping methods (cgSNP analysis and in silico MLVA typing) based on whole genome sequence (WGS) data were used to analyze a possible phylogenetic relationship among the members of our panel of isolates. We also used the in silico data to compare the results from lab-based MLVA with those of WGS based MLVA and also the reliability of MLVA based interpretations of the spread of outbreak strains with those based on WGS-cgSNP analysis.

Genotyping based on either WGS cgSNP analysis or in silico MLVA resulted both in ordering the panel of isolates into three main clusters though with quite different resolving capacity. The SDI determined for cgSNP analysis was 0.9 while it was only 0.56 for MLVA. While the former value would probably be considered as good, the latter one is moderate at best [35]. Genotyping based on differences in the fragment length of VNTR-markers or in single cgSNP difference allows for more accurate differentiation of microorganisms on the isolate level [19]. Nonetheless, both methods do not reflect the reality of epidemiological relations between outbreaks of a disease and their related outbreak strains if data are not interpreted on the background of epidemiological metadata. Their inclusion is a prerequisite for any meaningful interpretation of genotyping data to the purpose of defining the source and tracking the spread of an outbreak strain. Moreover, in case of the VNTR data used for 16-marker MLVA [31], one need to keep in mind that the markers used for analysis are composed of three panels. Panel 2a and 2b consist of microsatellites including some highly mutable repeat sequences where the number can be directly translated into a copy number of repeats [30,31]. Indeed, we found the highest diversity of alleles in markers Bruce07 (five alleles) and Bruce04, 16, 18 and 30 (three alleles each) which represent microsatellites. These markers mainly caused the differences in the cluster analysis for the 16-marker MLVA, as it was also described for *B. melitensis* by Shevtsova et al., 2019 [36]. Isolates with differences in VNTR-markers could be considered to represent a different outbreak strain or, the other way round, isolates with identical MLVAGT have been considered the same outbreak strain. In case the latter are found at different places this has been interpreted as spread of an outbreak strain between different locations [20,21,23,34]. However, as has been published before, highly mutable microsatellites are prone for homoplasy [30] and a given genotype might occur more than once at different places or even during subculturing in the laboratory. The addition and deletion of one repeat unit in Bruce07 of *B. abortus* S19 and RB51 vaccine strains after ten passages has been observed [20,37]. Hence, epidemiological interpretations based on microsatellites are not reliable, as will be discussed below. Omitting the highly homoplastic VNTR markers from a cluster analysis may increase the phylogenetic meaning of such an analysis and can be helpful to interpret the general regional relationships of *Brucella* spp. worldwide [38]. The other panel included in the MLVA-16 consists of minisatellites with varying sequences between the primer binding sites [30]. Some of them, like Bruce11, 12, 42, 55 are highly polymorphic, with extensive internal variations in the repeats, preventing the calculation of a consensus sequence and making calculation of the copy number of repeats usable for cluster analysis rather artificial. For the differentiation within clusters of our panel of isolates these markers do not play an important role as they all are mono-allelic except Bruce43 (two alleles).

While in our cgSNP analysis a single SNP defines a different GT one would not expect this deviation as a reliable definition to separate outbreak strains. The latter is not a bioinformatics but an epidemiological definition. Appropriately, one would like to define a certain number of SNPs to differentiate between outbreak strains and, hence, outbreaks. However, such a definition may be dependent on the lifestyle of an organism dealt with and the actual panel of isolates included in the analysis. In our panel we found some interesting correlations between epidemiological metadata and the number of SNP-differences.

The cgSNP based MP-tree in Figure 2 orders the 47 isolates and the two vaccine strains in three main clusters where cluster 2 is divided in subcluster 2a and 2b. Obviously, 20 members of cluster 2b are highly related showing a maximum distance of two cgSNPs in between, with the exception of HEA89 (six cgSNPs), though isolated from eight different governorates over a period of nine years (2012–2020). This period extends to even 15 years when additionally considering isolate HEA68 from 2006. This isolate has an identical cgSNPGT with 13 other animal isolates in this group, collected in 2012, 2013, 2014, and 2017 from four different governorates (Alexandria, Beheira, Dakahlia, and Sharqia). It differs with one cgSNP only to an isolate from Monufia (2019), to the human isolates from 2020 and to an isolate from Dakahlia (2013) and with two cgSNPs to an isolate from Ismailia (2017). These highly related GTs seems to reside in Egypt as an outbreak strain since a rather long time which has spread all over the northern part of Egypt. As our collection starts with a first isolate in 2006 there is no reason not to expect that this outbreak strain has been present before already. Interestingly, both the human isolates from Giza in 2020 also belong to this cluster, with only one cgSNP difference to the main group. However, we need to remain it open where the human cases originated from, eventually. The MST in Figure 3 defines isolates SH21, 15697, HEA37, HEA89 and the human isolates 21878, 21881 as descendants of the main cgSNPGT in subcluster 2b where the oldest isolates, HEA68 also belongs to. The homogenous group (7432-HEA140) of isolates clustering in 2a differ from this ancient GT by ten cgSNPs. Among them are also one isolate from a dog and one isolate from a cat, all from Damietta, sharing the same cgSNPGT like a cow from Damietta and another from Dakahlia (50 km distance). As all of them were isolated in 2015 from only two locations in northern Egypt (Damietta, Dakahlia) and as they differ in two cgSNPs maximum, they might demonstrate a separate introduction of *B. abortus* from abroad, maybe from the same infection source, having spread into the two governorates. The likelihood of outbreaks caused by recently acquired *B. abortus* is supported by a highly variable group of five isolates (HEA155-HEA161) in subcluster 2a comprising strains isolated in 2015, 2017 and 2018 with a distance of four and up to twelve cgSNPs. While it is tempting to speculate that the two isolates from Beheira (HEA113 and HEA118 from 2015) might closely be related to the isolates in SC2a from Damietta and Dakahlia from the same year, the rest of isolates in this subcluster would rather be considered outbreak strains with a different epidemiological background.

It should be highlighted that, with the exception of isolates HEA89 and HEA164, none of the members of cgSNP subclusters 2a and 2b could be differentiated by MLVA. This strongly argues against the hypothesis that identical MLVAGTs are an indication for identical outbreak strains and, even more important, that identical MLVAGTs found at different locations are, by themselves, a hint for the spread of an outbreak strain.

The six isolates in cluster 1 of the cgSNP analysis might represent the spread of a recently introduced outbreak strain over a long distance. This strain was isolated in 2020 from cattle in Beheira and Sharqia and from a sheep in Aswan as well, about 1000 km away from Beheira in the south of Egypt. The isolates represent three cgSNPGTs with only one and two cgSNPs among them. Hence, the MST in Figure 3 shows the six isolates in cluster 1 as an evolving outbreak strain with the three identical isolates as the probable ancestor. It is important to note that isolate 21906 from Aswan represents a separate genotype also in the MLVA. It is separated from the otherwise identical isolates in cluster 1 (Figure 4) by two copies of an 8 bp repeat of marker Bruce07. Qualifying this isolate as a different MLVAGT raises the question of whether or not one should be considered as a separate outbreak strain. Bruce07 belongs to the highly mutable markers where the copy number of the repeats may change quickly in both directions. The difference of one cgSNP in the entire core genome, however, characterizes the isolate from Aswan as highly related to all other isolates in cluster 1. Appropriately one can consider the Aswanian isolate the same outbreak strain as the isolates from Beheira/Sharqia. A similar issue occurs with HEA45 (SC3), an RB51 related field isolate with two SNPs difference to the vaccine 5842 and one repeat difference in the copy number of Bruce07. As HEA45 was isolated in the same year and from the same location as three more isolates identical in cgSNPGT it would not be considered a different outbreak strain.

Isolates in cluster 3 in both analyzes (Figure 2 and Figure 4) represents RB51 vaccine related strains. Isolates 5842 and 5843 are from vaccine batches purchased from the same company in 2014 though it is not known if two different batches were used. These two isolates differ in four cgSNPs, which might be a consequence of production of different vaccine batches. If so, it provides an estimation of the possible rate of mutations during culture and passaging of a given strain of *B. abortus*. The isolation of seven RB51 related strains, six from abortive materials and one from a slaughtered cow, was surprising and will necessitate further investigations. The circumstances for isolate HEA23, slaughtered possibly after a positive Rose Bengal test, are not clear. According to personal communications, both vaccine strains S19 and RB51 were in use in Egypt from 2012–2020. RB51 was used at large scale, whereas S19 was not used as much and rather in small farms. The only presence of RB51 vaccine strains in our panel of isolates might be a consequence of sampling bias. In both the NJ-tree and the MP-tree isolate 15649 is clearly separated as a single genotype with 199 cgSNPs difference from cluster 1 in the cgSNP analysis and three and four markers difference from cluster 1 and 3 in MLVA, respectively. The strain was isolated from a buffalo in 2017, from Beni Suef, a governorate about 150 km south of Cairo. Its origin needs to be left open as it is not related to any other isolates from buffaloes present in subclusters 2a and 2b (Figure 2). Interestingly, this isolate matches in the MLVA profile with an isolate from Portugal (2006) from the GenBank database (Figure S3).

As so far, the database for diversity and distribution of *B. abortus* in Egypt is rather scarce we were interested to search for possible correlations of our panel of isolates with entries in the public databases (Figure S2). The most striking point is the only ten cgSNPs distance of the biggest cluster 2 with database entries from the UK. Both the new outbreak strains from 2020 in cluster 1 and the single isolate 15649 are only distantly (29 and 36 cgSNPs, respectively) related to entries from the USA. Nonetheless, it is worth mentioning that after combining our isolates with the entries of the SRA database the original differentiation of cluster 2 into 16 cgSNPGTs and its subclustering was leveled out as a consequence of the generation of a new core genome. Hence, the distances in Figure S2 are not directly comparable with those in Figure 2.

According to the in silico MLVA (Figure S3), isolates in cluster 2 have the closest correlation to an entry from Argentina and isolates in cluster 1 have two markers difference to an entry from Spain. Most strikingly, isolate 15649 has the same MLVAGT as one strain from Portugal and other entries from the USA, Argentina and India differ in one marker.

Because of the fewer entries with reads in the SRA database usable for cgSNP analysis than entries with contigs from GenBank usable for MLVA and, as both sets of entries do not overlap, with the exception of a single isolate from Bangladesh, these both analyzes shown in Figures S2 and S3 cannot directly be compared with each other. For instance, the close relatives of isolate 15649 from Portugal and India found in the MLVA (Figure S3) were not included in the overall cgSNP analysis (Figure S2). Hence, it is not possible to verify the MLVA based relationship for this strain by cgSNP analysis. A remarkable outcome of the comparisons with the entries from the databases was the detection of RB51 vaccine like strains isolated in the USA and India.

Interesting in this context is the record of the Food and Agriculture Organization of the United Nations (FAOSTAT) on the import of animals for breeding or slaughtering purposes from several countries to Egypt. The downloaded excel sheet from FAOSTAT (http://www.fao.org/faostat/en/#data/TM, accessed on 17 July 2021) is provided in the Supplementary Material (Table S10). Animals were imported from UK in 1993 (cattle), from the USA in 1987–2019 (cattle, buffaloes) and from Portugal in 2001 (cattle). Further entries are from Spain and India. Imported cattle from USA were imported for breeding, while the animals imported from UK, Spain, Portugal and India were imported for slaughtering. The isolates in cluster one were isolated in 2020 but cattle were imported from the UK in 1993. The isolates in cluster two were all isolated in 2006–2020, but the import from the USA took place in 1987–2019. Isolate 15649 was isolated in 2017 but cattle were imported from Portugal in 2001. Based on these data, a possible source of infection from these mentioned countries is not ruled out. However, it is still difficult to explain because the animals, except the ones from USA, were all imported for slaughtering reasons.

Data from a total of 83 *B. melitensis* and *B. abortus* samples were compared in order to verify the reliability of either the MLVA based on PCR fragment lengths or drawn in silico from contigs assembled by the bioinformatics pipeline. The deviations were re-checked by repetitive lab analyzes and sequencing of PCR fragments with some unambiguity after capillary electrophoresis. The results confirmed the fragment length of all Bruce markers as drawn from the in silico analysis. The lab-based MLVA may be highly error prone or may even not result in any interpretable data output [20,23]. Such errors do negatively influence the exchange of results between laboratories and the interpretation of epidemiological data like the spread of outbreak strains.

## 5. Conclusions

Genotyping of WGS data by whole core genome SNP analysis and in silico MLVA revealed the presence of a rather ancient outbreak related genotype of *B. abortus* in Egypt and some events of outbreaks caused by variable and, possibly, newly introduced strains of *B. abortus*. The cgSNP analysis provides a much higher resolution of genotypes than MLVA and, when correlated to the associated epidemiological metadata, allows for the differentiation of outbreaks by defining different outbreak strains. In this respect, MLVA data are error prone and can lead to wrong interpretations of outbreak events. Nevertheless, considering the limitations mentioned above, it may be useful to compare in silico MLVA results from new isolates with strains from previous outbreak studies for which data are available only as MLVA genotypes.

For a grave interpretation of our data collection some obvious limitations of the study should be taken into consideration. The majority of samples leading to isolates of *B. abortus* were collected in 2012–2020, except year 2016 and only one isolate was taken in 2006. The lack of isolates from 2007–2011 limits the possibility to calculate and interpret the spread and evolution of the ancient GT from 2006 until 2020. Also, GTs found more recently might have had a former relative in the years before 2012.

Moreover, there is a great sampling bias in the data collection as the majority of samples are from the north of Egypt, mainly from Dakahlia while other regions of the country are not represented at all. One should also keep in mind that the available metadata on the region of isolation does not reflect the exact location of sample collection. The similar issue applies for the human isolates as we do not know where the patients eventually came from.

## Data Availability

All supporting data are part of the publication. Fastq-files of the bioproject are available under: https://www.ncbi.nlm.nih.gov/bioproject/742519, accessed on 24 April 2021.

## Supplementary Materials

The following are available online at https://www.mdpi.com/article/10.3390/microorganisms9091942/s1, Table S1: Egyptian isolates and the reference strain 2308 with its metadata, Table S2: Genome characteristics of the Egyptian isolates, Table S3: Bruce-ladder primer, Table S4: cgSNP distance table of the Egyptian isolates based on the reference *B. abortus* 2308, Table S5: cgSNP distance table of the Egyptian isolates based on the reference *B. abortus* RB51, Table S6: MLVA results of the Egyptian isolates, Table S7: cgSNP distance table of entries from SRA database and the Egyptian isolates, Table S8: MLVA results of the database entries, Table S9: *B. abortus* isolates from SRA and GenBank with its metadata, Table S10: Downloaded data from FAOSTAT of imported animals from several countries to Egypt, Figure S1: MST based on MLVA data of the Egyptian *B. abortus* isolates, Figure S2: MST based on cgSNP data of the Egyptian isolates and the entries of SRA, Figure S3: MST based on MLVA data of the Egyptian isolates and the entries from SRA and GenBank, Figure S4: 2524 bp fragment detection of the Egyptian isolates classified as RB51, Supplementary Map: QGIS file-Map of *B. abortus* isolates.
