# Supplementary material for: Tracking the Distribution of Brucella abortus in Egypt Based on Core Genome SNP Analysis and In Silico MLVA-16
Source: Microorganisms. 2021 Sep 13;9(9):1942. doi: 10.3390/microorganisms9091942 (PMC8469952; doi:10.3390/microorganisms9091942)
Supplement: Supplementary file 1 [file microorganisms-09-01942-s001.zip › Figure S1 MST MLVA based of the Egyptian B. abortus isolates.pdf]

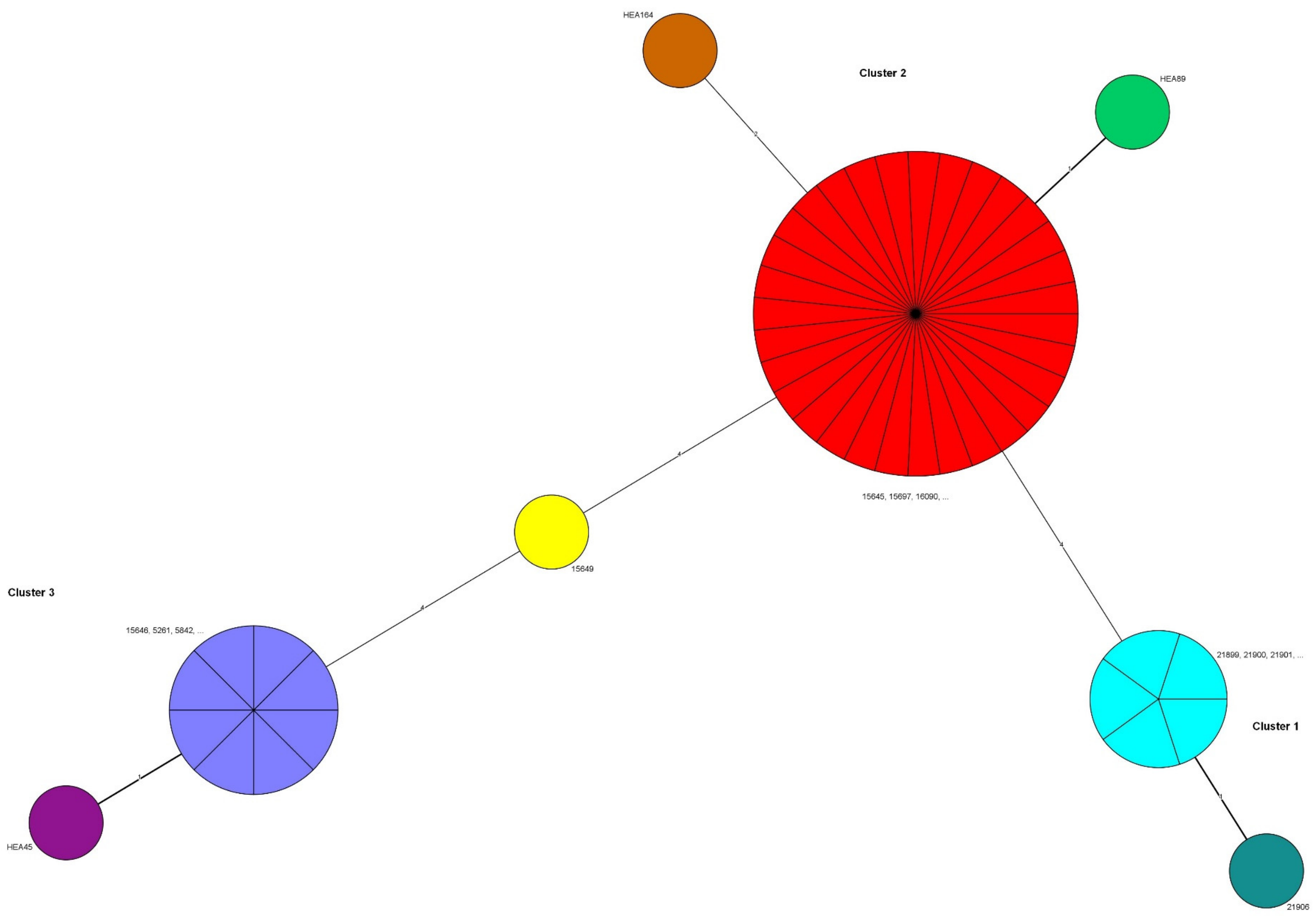

Figure S1. Minimum spanning tree (MST) for the 45 *B. abortus* field isolates plus two human isolates and two vaccine batches based on in silico MLVA. The same color represents the same genotype. Circles represent MLVA-16 genotypes and the size of the circle indicates the number of strains with that genotype.
