# Supplementary material for: Tracking the Distribution of Brucella abortus in Egypt Based on Core Genome SNP Analysis and In Silico MLVA-16
Source: Microorganisms. 2021 Sep 13;9(9):1942. doi: 10.3390/microorganisms9091942 (PMC8469952; doi:10.3390/microorganisms9091942)
Supplement: Supplementary file 1 [file microorganisms-09-01942-s001.zip › Figure S2 MST SNP with public entries.pdf]

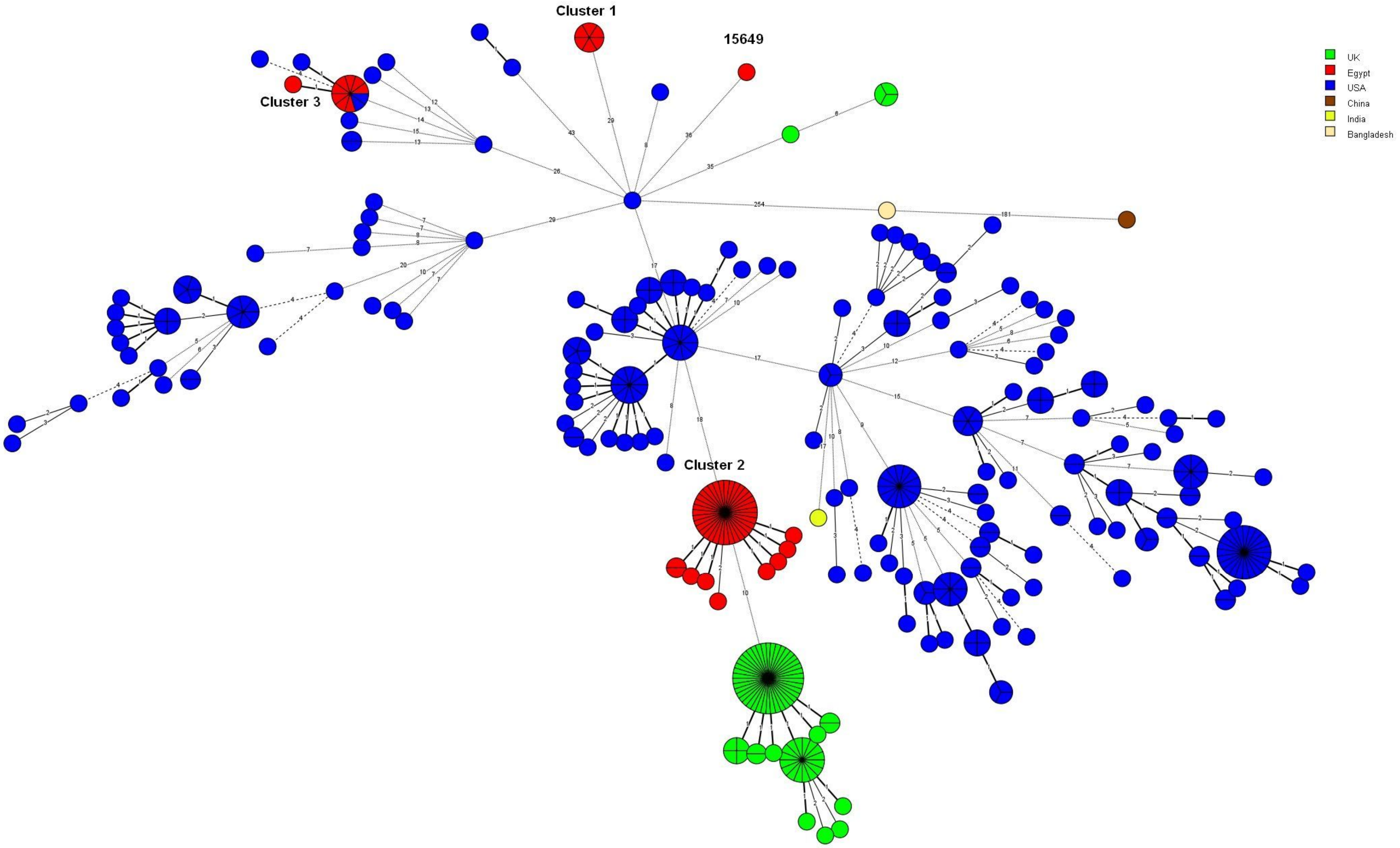

Figure S2. MST based on SNP analysis generated from the 47 isolates from Egypt used in this study, both vaccine batches and the 364 available international entries. The same color represents the same genotype. *B. abortus* 2308 was set as reference genome. The isolates marked in red are the ones from Egypt. The numbers inserted in the picture represent the different clusters and isolate 15649, as shown in Figure 2 and 4.
