# Supplementary material for: Tracking the Distribution of Brucella abortus in Egypt Based on Core Genome SNP Analysis and In Silico MLVA-16
Source: Microorganisms. 2021 Sep 13;9(9):1942. doi: 10.3390/microorganisms9091942 (PMC8469952; doi:10.3390/microorganisms9091942)
Supplement: Supplementary file 1 [file microorganisms-09-01942-s001.zip › Figure S3 MLVA with public entries.pdf]

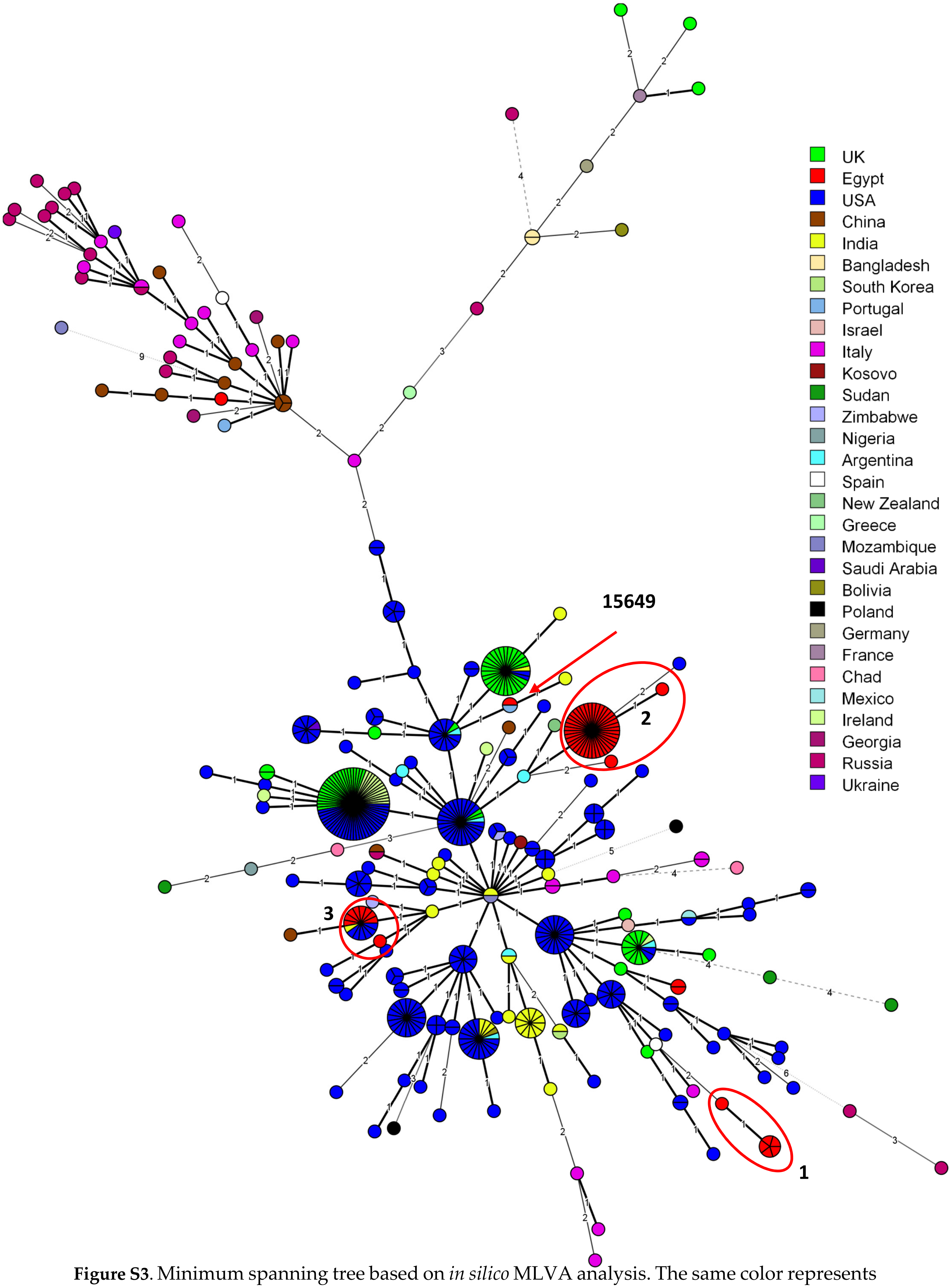

Figure S3. Minimum spanning tree based on *in silico* MLVA analysis. The same color represents the same genotype. The strains isolated from Egypt are marked in red and highlighted by circles. The inserted numbers in this picture represents the different clusters shown in Figure 2 and Figure 4.
