# Supplementary material for: Tracking the Distribution of Brucella abortus in Egypt Based on Core Genome SNP Analysis and In Silico MLVA-16
Source: Microorganisms. 2021 Sep 13;9(9):1942. doi: 10.3390/microorganisms9091942 (PMC8469952; doi:10.3390/microorganisms9091942)
Supplement: Supplementary file 1 [file microorganisms-09-01942-s001.zip › Figure S4 fragment detection of the RB51 isolates.pdf]

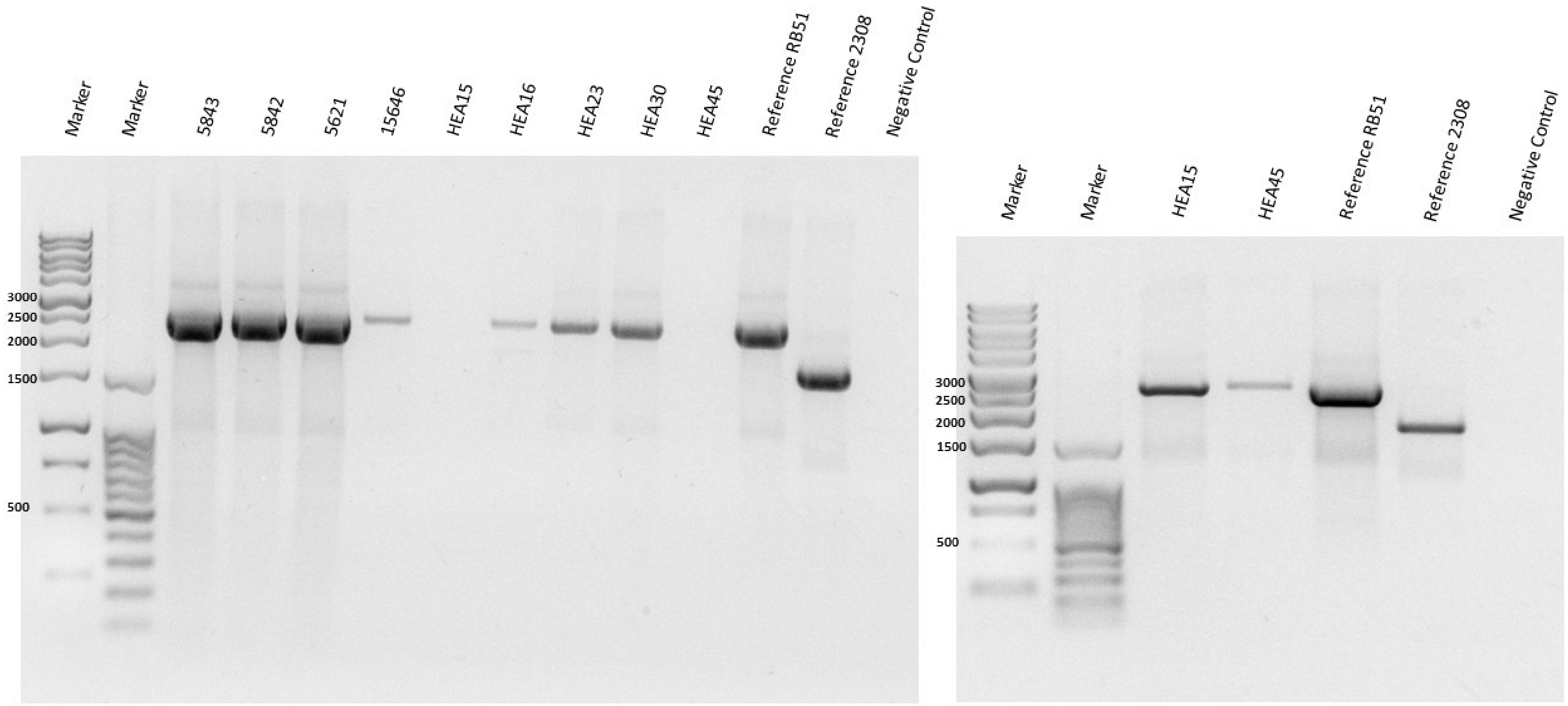

Figure S4. 1% agarose gel electrophoresis for the 2524 bp fragment detection of the isolates classified as RB51 strains with the primers BMEI0998f & BMEI0997r. Since the isolates HEA15 and HEA45 showed no fragments in the left gel, the PCR was repeated, showing the results in the right gel. The DNA ladder is indicated in base pairs (bp).
