# Supplementary material for: Tracking the Distribution of Brucella abortus in Egypt Based on Core Genome SNP Analysis and In Silico MLVA-16
Source: Microorganisms. 2021 Sep 13;9(9):1942. doi: 10.3390/microorganisms9091942 (PMC8469952; doi:10.3390/microorganisms9091942)
Supplement: Supplementary file 1 [file microorganisms-09-01942-s001.zip › Table S3 Bruceladder PCR Primer.pdf]

Table S3: Primer used for the Bruce-ladder PCR and its fragment lengths

BMEI0998f	ATCCTATTGCCCCGATAAGG	1682 bp fragment
BMEI0997r	GCTTCGCATTTTCACTGTAGC	
BMEII0843f	TTT ACA CAG GCA ATC CAG CA	1071 bp fragment
BMEII0844r	GCG TCC AGT TGT TGT TGA TG	
BMEII0721f	CCA ACC GTA TGT CCT CTC T	766 bp fragment
BMEII0722r	TGC GGG AAC TGG TGT TCG ACG	
BMEII0428f	GCC GCT ATT ATG TGG ACT GG	587 bp fragment
BMEII0428r	AAT GAC TTC ACG GTC GTT CG	
BMEI0535f	GCG CAT TCT TCG GTT ATG AA	450 bp fragment
BMEI0536r	CGC AGG CGA AAA CAG CTA TAA	
BMEIr02f	CTA CTC AAG GAC AAC AGG TG	344 bp fragment
BMEIr02f	TGT GTC GTT TAA GGC AAT AGG	
BR0953f	GGA ACA CTA CGC CAC CTT GT	272 bp fragment
BR0953r	GAT GGA GCA AAC GCT GAA G	
BMEI0752f	CAG GCA AAC CCT CAG AAG C	218 bp fragment
BMEI0752r	GAT GTG GTA ACG CAC ACC AA	
BMEII0987f	CGCAGACAGTGACCATCAAA	152 bp fragment
BMEII0987r	GTATTCAGCCCCCGTTACCT	
